# Research on the Principle and Cooperative Processing Method of MRS Multisystem Joint Detection

**DOI:** 10.3390/s21206725

**Published:** 2021-10-10

**Authors:** Cong Li, Zhaofa Zeng, Zhuo Wang, Xiaofeng Yi

**Affiliations:** 1 College of Geo-Exploration Science and Technology, Jilin University, Changchun 130026, China; congli19@mails.jlu.edu.cn (C.L.); zengzf@jlu.edu.cn (Z.Z.); zwang20@mails.jlu.edu.cn (Z.W.); 2 Key Laboratory of Applied Geophysics, Ministry of Land and Resources, Jilin University, Changchun 130026, China; 3 College of Instrumentation & Electrical Engineering, Jilin University, Changchun 130026, China; 4 Key Laboratory of Geo-Exploration Instrumentation, Ministry of Education, Jilin University, Changchun 130061, China

**Keywords:** magnetic resonance, dual-system coordination, groundwater detection, work efficiency

## Abstract

Magnetic resonance sounding (MRS) technology is the only geophysical means to directly and quantitatively detect groundwater and has achieved good results in hydrogeological prospecting applications. In recent years, researchers have conducted considerable research on the efficiency of a single instrument, yielding certain results. However, the overall work efficiency of this method has not been effectively determined in its application to a large-scale survey. Hence, we propose both a joint detection method for MRS that determines the minimum working distance when multiple systems operate simultaneously and a collaborative measurement method of dual systems operating simultaneously in a fixed range of work areas. The cooperative working mode of the instruments is tested in the detection area, and the working mode proposed in this paper is shown to effectively avoid measurement interference between systems. Compared with the working mode of a single set of instruments, the measurement efficiency is more than doubled. Through this research, the feasibility of multiple MRS instruments working together in the same work area is verified, which provides effective technical support for the rapid and high-efficiency utilization of MRS over a wide range of measurement areas.

## 1. Introduction

Magnetic resonance sounding (MRS) technology enables the direct detection of groundwater. Since the successful development of the detection system, it has been utilized in engineering applications in Russia, France, the United States, Germany, China, and other countries [1,2,3,4,5,6,7,8] and achieved good application effects [9,10,11,12]. In the process of large-scale groundwater detection and evaluation, the detection efficiency is an important factor affecting the speed of groundwater evaluation. Scientists at home and abroad have proposed different solutions in terms of detection instruments and measurement methods to improve the efficiency of the detection system.

At present, researchers mainly reduce the single measurement time to improve measurement efficiency. Peshkovsky et al. [13] adopted a new type of active ringing suppression circuit, which is inductively coupled to the main coil through a low-inductance series transformer that is conveniently placed, greatly shortening the recovery time of the probe. This method improves the single signal acquisition time and work efficiency. To shorten the measurement dead time problem, the GeoMRS instrument and multichannel surface MRS application developed by Vista Clara of the United States use the multichannel reception measurement mode to significantly shorten the measurement dead time by 10 ms [7], further compressing the single signal acquisition time. Li et al. [14] proposed the concept of switching circuits through Q-toggle circuits to shorten the dead time by 12.4 ms. Behroozmand et al. [15] used a central loop configuration, which employs a shielded receiving coil to potentially reduce the dead time of the instrument to almost zero. At the same time, some researchers have proposed improving the measurement speed by changing the detection sequence. Grunewald et al. [16] used the adiabatic pulse method to replace or supplement the standard single-frequency emission resonance pulse to increase the signal amplitude to shorten the measurement time. Zhang [17] proposed a working method based on matching energy storage capacitors, which can achieve a measurement sequence of multiple emissions with one charge. The average single transmission and data acquisition time is reduced from 10.54 to 2.75 s, and its work efficiency is increased fourfold.

As mentioned above, the methods to improve efficiency all offer improvements when measuring with a single instrument. At present, the fastest working speed of a single instrument still requires more than 20–30 min to complete the task, and the measurement time may be longer under the influence of noise. Therefore, in the process of large-area survey area exploration, two or three instruments are usually used to carry out work at the same time to significantly improve the detection efficiency. In the magnetic resonance sounding method, because the detection system needs to receive nanovolt magnetic resonance signals, when multiple instruments work at the same time, mutual interference will inevitably affect their detection performance. At present, there is no public literature discussing and researching the linear distance between two instruments necessary for them to operate without interference and whether the cooperative working mode can be adapted to enable the two instruments to work simultaneously in a small space.

To solve this problem, we propose the detection of electromagnetic interference and coupling characteristics between antennas when two instruments are working at the same time. By carrying out theoretical calculations of electromagnetic coupling and taking the minimum resolution of the detection system as the constraint, we determine the shortest linear distance at which two instruments can work without interference. Moreover, a detection mode with two systems working together is proposed, which enables multiple detection systems to perform noninterference detection at the same time in a small detection area, and the working efficiency of magnetic resonance sounding technology is significantly improved. This method provides a technical reference for the large-scale application of magnetic resonance sounding detection methods.

## 2. The Theory of the Magnetic Resonance Sounding Method Detection

Magnetic resonance sounding is a geophysical method that can be used to directly detect groundwater [10]. It uses a transmitting antenna to form an artificially exciting electromagnetic field to generate a magnetic resonance sounding effect for hydrogen protons in groundwater. At the same time, it uses a receiving antenna to capture the MRS generated by hydrogen protons in the water. By analyzing the characteristic parameters of the signals, one can determine the existence of groundwater.

**Figure 1:**
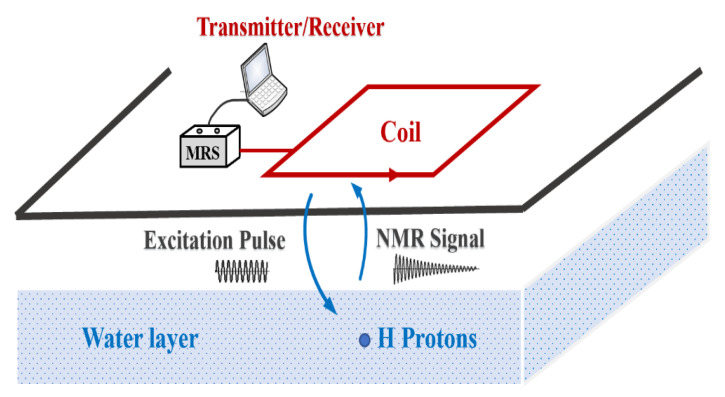
Schematic diagram of the magnetic resonance sounding measurement [1].

The basic principle of magnetic resonance sounding detection is shown in Figure 1. Among them, hydrogen protons in water undergo Larmor precession under the action of a stable geomagnetic field $B_{0}\;$ [1]. The Larmor frequency is related only to the geomagnetic field frequency of the detection site. To make the hydrogen protons transition to the adjacent Zeeman energy level in a steady state, a coil placed on the ground generates an alternating current to form an excitation magnetic field, making the excitation frequency the same as the Larmor frequency. After a hydrogen proton is excited by magnetic resonance, it transitions to a higher energy level. At this time, the emission of the alternating magnetic field is stopped, and then the hydrogen protons gradually release energy to return to a steady state. The released energy quantum appears macroscopically as an MRS with a Larmor frequency and is received by the same coil. By analyzing the characteristic parameters of the MRS, one can infer the hydrological information of the detection site [18,19,20].

After a large number of hydrogen protons transition to a high energy level, the emission of the alternating magnetic field is stopped, and then the hydrogen protons gradually release energy and return to a stable state. The released energy quantum is macroscopically expressed as an MRS with a Larmor frequency. When the coil is used to receive the signal, the hydrological information of the detection site can be inferred. The magnitude of the signal represents the number of hydrogen protons. The time-domain expression of the magnetic resonance signal for different transmit pulse moments *q* is shown in Equation (1) [21].

(1) $$E\left(t,q\right)=E_{0}\left(q\right)exp\left(-\frac{t}{T_{2}^{*}}\right)cos(\omega_{0}t+\varphi_{0})$$

Among them, *E*_0_*(q)* is the initial amplitude of the MRS at different transmit pulse moments [20], *T*_2_* is the average lateral decay time [22,23,24], and *φ*_0_ is the initial phase of the signal [25]. Three key parameters of the MRS exist, i.e., the water content, pore size, and electrical conductivity of the aquifer per unit volume within the detection range. In the process of magnetic resonance sounding detection, the initial amplitude *E*_0_ obtained by the received signal is at the nV level. However, the excitation pulse moment q needs to reach approximately 8000 Ams to meet the detection depth requirement; at this time, the corresponding emission current can reach approximately 200 A [26]. Therefore, when more than one instrument operates at the same time, one instrument system and another instrument system will inevitably produce mutual electromagnetic interference, which will affect the detection performance. By increasing the linear distance between the instruments and using the attenuation characteristics of electromagnetic waves in an air medium, one can reduce the mutual interference between systems and realize the interference-free operation of multiple instruments. Determining the distance requires a numerical calculation of the related electromagnetic coupling.

## 3. Calculation of the Electromagnetic Field Emission and Reception Response of a Magnetic Resonance Sounding Antenna

When using the magnetic resonance sounding method to measure groundwater on the surface, the antenna is generally laid in a horizontal direction, and more than one instrument is detected at the same time, after which electromagnetic interference occurs between the antennas. When the current in two adjacent coils changes, a mutual induction electromotive force is generated in each coil. The mutual electromotive force in one coil is related not only to the speed of the current change in the other coil but also to the structure of the two coils and their positions relative to one another.

To evaluate the electromagnetic interference between antennas, it is necessary to calculate the spatial magnetic field distribution of the current-carrying coil. According to classical theory [27,28], the rectangular current-carrying coil is regarded as four straight current-carrying wires. The wires are calculated in sections and then superimposed to obtain the spatial magnetic field distribution of the rectangular current-carrying coil. Next, according to the actual situation of field detection, that is, when the signal frequency and the maximum current are determined, the relationship between the induced electromotive force of the coaxial equal square coil and the mutual distance between the two coils is calculated. Then, according to the actual situation of field detection, that is, when the signal frequency and maximum current are determined, the relationship between the induced electromotive force of the coaxial square coils of equal size and the mutual distance between the two coils is calculated.

**Figure 2:**
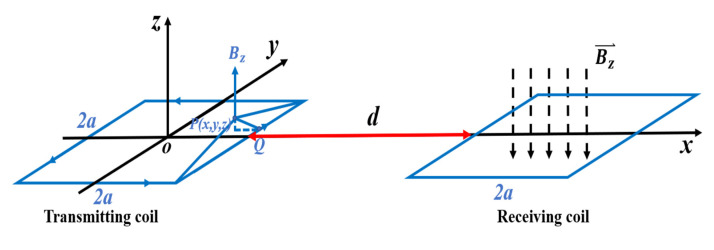
The magnetic field of the transmitting coil T and the receiving coil R [29].

The relative position of the transmitting coil T and the receiving coil R on the ground is shown in Figure 2. The side length of the two coils is 2a, and the distance between the adjacent sides is d. The transmitting coil T is regarded as four long, straight wires connected together with a current I and a length of 2a. It is placed in a rectangular coordinate system OXYZ, where the center of the square is taken as the coordinate origin and the axis is the *z*-axis. The magnitude of the magnetic induction intensity *B* generated by the transmitting coil T at any point p in the *z*-axis direction over time is as follows [29,30]:

(2) $$\begin{array}{c} B=(\frac{a+x}{\sqrt{{\left(a+x\right)}^{2}+{\left(a+y\right)}^{2}+z^{2}}}+\frac{a-x}{\sqrt{{\left(a-x\right)}^{2}+{\left(a+y\right)}^{2}+z^{2}}}).\\ +\frac{\mu_{0}I\left(t\right)a-y}{4\pi \left[{\left(a-y\right)}^{2}+z^{2}\right]}(\frac{a-x}{\sqrt{{\left(a-x\right)}^{2}+{\left(a-y\right)}^{2}+z^{2}}}+\frac{a+x}{\sqrt{{\left(a+x\right)}^{2}+{\left(a-y\right)}^{2}+z^{2}}}).\\ +\frac{\mu_{0}I\left(t\right)a-x}{4\pi \left[{\left(a-x\right)}^{2}+z^{2}\right]}(\frac{a+y}{\sqrt{{\left(a-x\right)}^{2}+{\left(a+y\right)}^{2}+z^{2}}}+\frac{a-y}{\sqrt{{\left(a-x\right)}^{2}+{\left(a-y\right)}^{2}+z^{2}}}).\\ +\frac{\mu_{0}I\left(t\right)a+x}{4\pi \left[{\left(a+x\right)}^{2}+z^{2}\right]}\left(\frac{a-y}{\sqrt{{\left(a-x\right)}^{2}+{\left(a+y\right)}^{2}+z^{2}}}+\frac{a+y}{\sqrt{{\left(a+x\right)}^{2}+{\left(a+y\right)}^{2}+z^{2}}}\right). \end{array}$$

In the field experiment, the coil is laid on the ground so that *z* = 0. In the coil plane, that is, the horizontal OXY plane, the formula of the change in magnetic induction intensity $B_{z0}$ with time can be obtained as shown below:

(3) $$\begin{array}{r} B_{z0}\left(t\right)=\frac{\mu_{0}I\left(t\right)}{4\pi a+y}\left(\frac{a+x}{\sqrt{{\left(a+x\right)}^{2}+{\left(a+y\right)}^{2}}}+\frac{a-x}{\sqrt{{\left(a-x\right)}^{2}+{\left(a+y\right)}^{2}}}\right).\\ +\frac{\mu_{0}I\left(t\right)}{4\pi a-y}\left(\frac{a-x}{\sqrt{{\left(a-x\right)}^{2}+{\left(a-y\right)}^{2}}}+\frac{a+x}{\sqrt{{\left(a+x\right)}^{2}+{\left(a-y\right)}^{2}}}\right).\\ +\frac{\mu_{0}I\left(t\right)}{4\pi a-x}\left(\frac{a+y}{\sqrt{{\left(a-x\right)}^{2}+{\left(a+y\right)}^{2}}}+\frac{a-y}{\sqrt{{\left(a-x\right)}^{2}+{\left(a-y\right)}^{2}}}\right).\\ +\frac{\mu_{0}I\left(t\right)}{4\pi a+x}\left(\frac{a-y}{\sqrt{{\left(a+x\right)}^{2}+{\left(a-y\right)}^{2}}}+\frac{a+y}{\sqrt{{\left(a+x\right)}^{2}+{\left(a+y\right)}^{2}}}\right). \end{array}$$

The magnetic flux passing through the receiving coil R is expressed as $d\varphi =B_{z0}\left(t\right)d_{s}$ [27]; then, the total magnetic flux passing through the receiving coil R is:

(4) $$\varphi =\frac{\mu_{0}I\left(t\right)}{4\pi }\int_{a+d}^{3a+d}\int_{-a}^{a}[\begin{array}{r} \frac{1}{a+y}\left(\frac{a+x}{\sqrt{{\left(a+x\right)}^{2}+{\left(a+y\right)}^{2}}}+\frac{a-x}{\sqrt{{\left(a-x\right)}^{2}+{\left(a+y\right)}^{2}}}\right)\\ +\frac{1}{a-y}\left(\frac{a-x}{\sqrt{{\left(a-x\right)}^{2}+{\left(a-y\right)}^{2}}}+\frac{a+x}{\sqrt{{\left(a+x\right)}^{2}+{\left(a-y\right)}^{2}}}\right)\\ +\frac{1}{a-x}\left(\frac{a+y}{\sqrt{{\left(a-x\right)}^{2}+{\left(a+y\right)}^{2}}}+\frac{a-y}{\sqrt{{\left(a-x\right)}^{2}+{\left(a-y\right)}^{2}}}\right)\\ +\frac{1}{a+x}\left(\frac{a-y}{\sqrt{{\left(a+x\right)}^{2}+{\left(a-y\right)}^{2}}}+\frac{a+y}{\sqrt{{\left(a+x\right)}^{2}+{\left(a+y\right)}^{2}}}\right) \end{array}]d_{x}d_{y}.$$

Let $A=\int_{a+d}^{3a+d}\int_{-a}^{a}[\frac{1}{a+y}\left(\frac{a+x}{\sqrt{{\left(a+x\right)}^{2}+{\left(a+y\right)}^{2}}}+\frac{a-x}{\sqrt{{\left(a-x\right)}^{2}+{\left(a+y\right)}^{2}}}\right)+\frac{1}{a-y}(\frac{a-x}{\sqrt{{\left(a-x\right)}^{2}+{\left(a-y\right)}^{2}}}+\frac{a+x}{\sqrt{{\left(a+x\right)}^{2}+{\left(a-y\right)}^{2}}})+\frac{1}{a-x}\left(\frac{a+y}{\sqrt{{\left(a-x\right)}^{2}+{\left(a+y\right)}^{2}}}+\frac{a-y}{\sqrt{{\left(a-x\right)}^{2}+{\left(a-y\right)}^{2}}}\right)+\frac{1}{a+x}(\frac{a-y}{\sqrt{{\left(a+x\right)}^{2}+{\left(a-y\right)}^{2}}}+\frac{a+y}{\sqrt{{\left(a+x\right)}^{2}+{\left(a+y\right)}^{2}}})]d_{x}d_{y}$; then, the magnetic flux passing through the receiving coil R is $\varphi =\frac{\mu_{0}I\left(t\right)}{4\pi }*A$. The induced electromotive force $\epsilon =-\frac{d\varphi }{dt}$ [26], $I=I_{0}\;cos\;\omega t$, and $\mu_{0}=4\pi *\frac{10^{-7}H}{m}$. Therefore:

$$\epsilon =-\frac{\frac{\mu_{0}I\left(t\right)}{4\pi }}{dt}*A=A*\frac{\mu_{0}}{4\pi }*\omega *I_{0}\;sin\;\omega t=A*10^{-7}*\omega \cdot I_{0}\;sin\;\omega t=A*10^{-7}*2\pi f*I_{0}\;sin\;\omega t.$$

**Figure 3:**
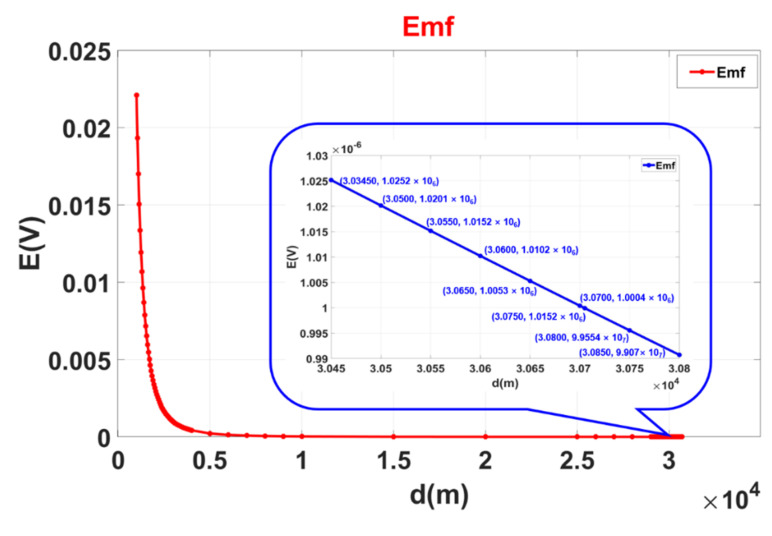
Relationship between the electromagnetic interference intensity of two detection systems and the distance.

According to the actual situation of the magnetic resonance sounding instrument, the maximum emission current of the instrument is 200 A, and the frequency is 2326 Hz. The distance between the two coils is increased to obtain the electromagnetic induction voltage trend of the receiving coil, and the result is shown in Figure 3.

The actual space noise in the field is generally 1000 nV. When the induced voltage of the receiving coil is less than the space noise, the induced voltage between the two coils can be ignored. Through calculation, when the distance between the centers of the two coils is 30.8 km, the induced voltage between the two coils is 1.0004 × 10^−6^ V, which is close to the spatial noise of 1000 nV; that is, when the distance between the two magnetic resonance sounding instruments is 30.8 km, they can work without interruption at the same time.

## 4. Discussion

**Figure 4:**
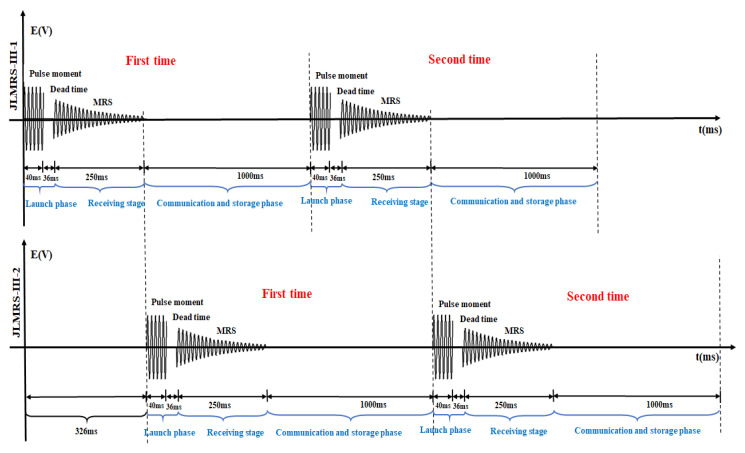
Alternating process of the working time of two magnetic resonance sounding instruments.

According to the above calculation, when the distance between the two magnetic resonance sounding instruments is 30.8 km, they can work without interruption at the same time. The different work areas are as follows:

(1) When the linear distance in the work area is much larger than 30.8 km, two magnetic resonance sounding instruments can be used uninterruptedly at the same time, that is, in free-detection mode, effectively reducing the field time and increasing the efficiency of fieldwork.
(2) When the linear distance of the work area is less than 30.8 km, the free-detection mode will inevitably cause mutual interference between the instruments, affecting the measurement performance due to the electromagnetic induction. To solve this problem, we propose a new mode of operation, that is, multiple instruments operating in an alternating fashion. The working process is as follows:

When a single instrument performs a single measurement, the excitation magnetic field is first generated by the transmitter module of the detection system, and it stops after 76 ms. This process includes the emission time and the dead time; then, the magnetic resonance sounding signal is received through the receiving module. The duration of this receiving process is 250 ms. Finally, the communication module stores the data, and the data processing time is 1000 ms. When two or even multiple instruments work together, it is necessary that the transmitting process and receiving process between the instruments do not interfere with each other. That is after one magnetic resonance sounding instrument completes the transmission process and the process of receiving the magnetic resonance sounding signal, another instrument performs the transmission and reception process. This time alternation process of two instruments working together is shown in Figure 4. The first magnetic resonance sounding instrument completes the transmission and reception process; that is, after 326 ms, the second magnetic resonance sounding instrument starts the transmission and reception process. This alternating operation can improve the work efficiency of two or more instruments in the field.

**Figure 5:**
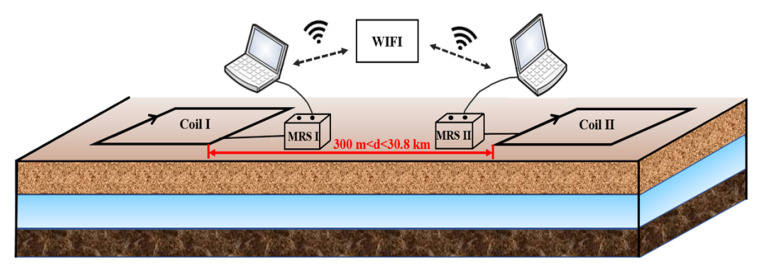
Schematic diagram of the communication mode of two sets of magnetic resonance sounding systems.

**Figure 6:**
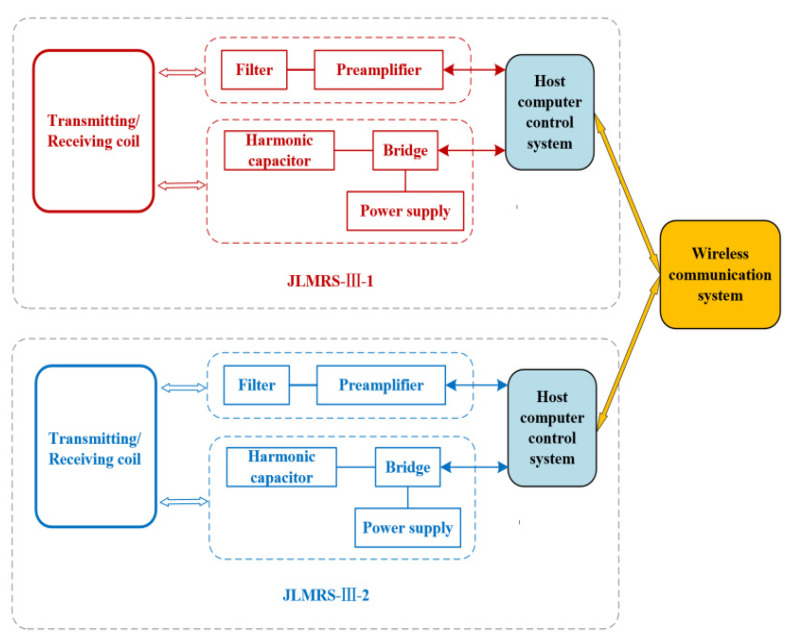
Block diagram of the detection system of two magnetic resonance sounding instruments.

To better solve the problem of alternating the operation of two instruments, it is proposed to establish a wireless communication system between the two instruments to ensure that the first instrument completes the transmission process in time before the other instrument performs the transmission process, resulting in better completion of the information interaction between the two instruments. A schematic diagram of the communication between the two magnetic resonance sounding systems is shown in Figure 5. The two instruments are placed together in the fieldwork area, and the distance between the two coils is greater than 1.5 times the side length; that is, the distance between their nearest sides is more than three times the side length. The two magnetic resonance sounding instruments exchange information through a wireless communication system. A system block diagram of the internal working of each instrument is shown in Figure 6, which includes the transmitting system, receiving system, upper computer control system, and wireless communication system of the two instruments. The wireless communication system is composed of industrial routers (model:), which can establish a long-distance WIFI network to ensure that the dual PC system can interactively access the marked files in the local area network and realize the dual-host coordinated control.

**Figure 7:**
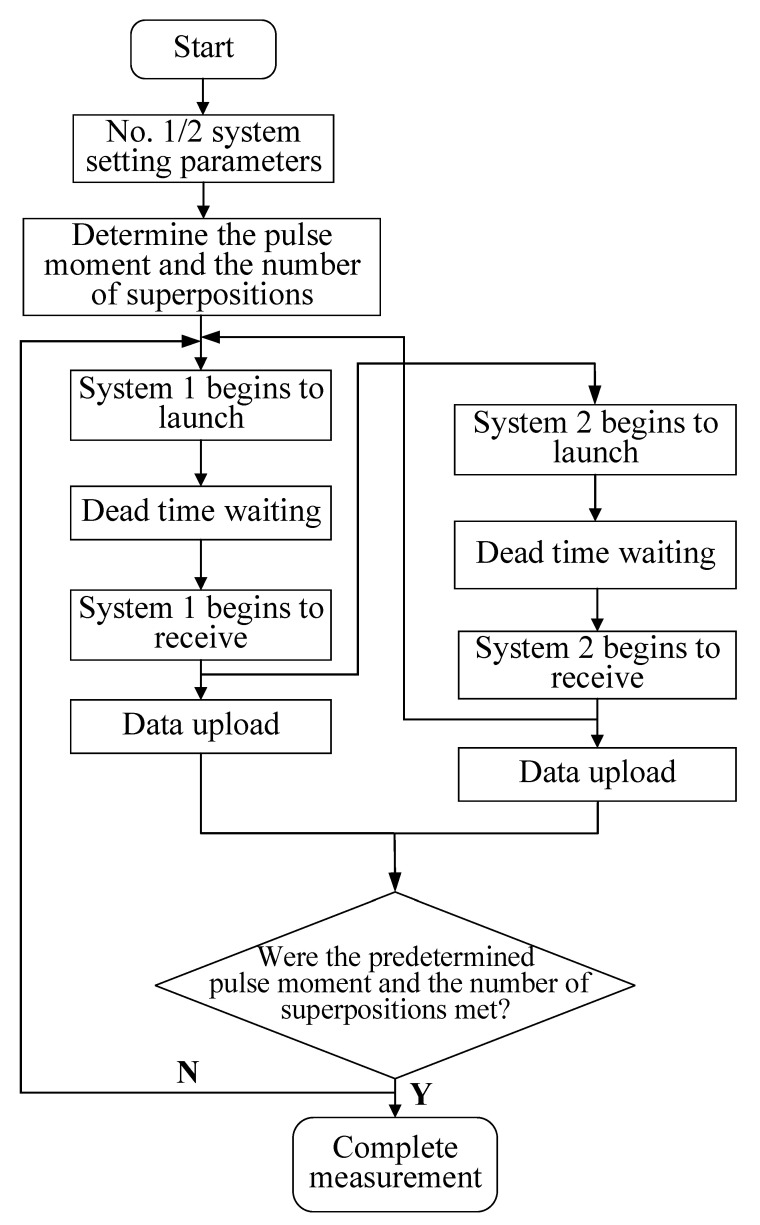
Diagram of the measurement process of the magnetic resonance sounding dual-machine detection system.

The measurement process of the two sets of magnetic resonance sounding instrument detection systems shown in Figure 7 is mainly divided into two stages.

(1) The first magnetic resonance sounding instrument starts to transmit the pulse sequence through the system settings and begins to receive MRSs signals after the dead time. After completion, the host computer control system transmits the “received” message to the wireless communication system. Then, the message is transmitted to the upper computer control system of the second magnetic resonance sounding instrument through the wireless communication system, and instruction is emitted to start the measurement.
(2) Then, the second magnetic resonance sounding instrument starts the transmission process through the system settings. After receiving the MRS, the “received” message is transmitted to the wireless communication system through the upper computer control system and then sent to the upper computer control system of the first magnetic resonance sounding instrument. When the first magnetic resonance sounding instrument completes the first measurement, the host computer control system sends an instruction to start the second measurement.
(3) The above process is repeated until all excitation pulse moments are completed to realize alternate and effective detection from the surface to the depth of the underground target.

## 5. Results

**Figure 8:**
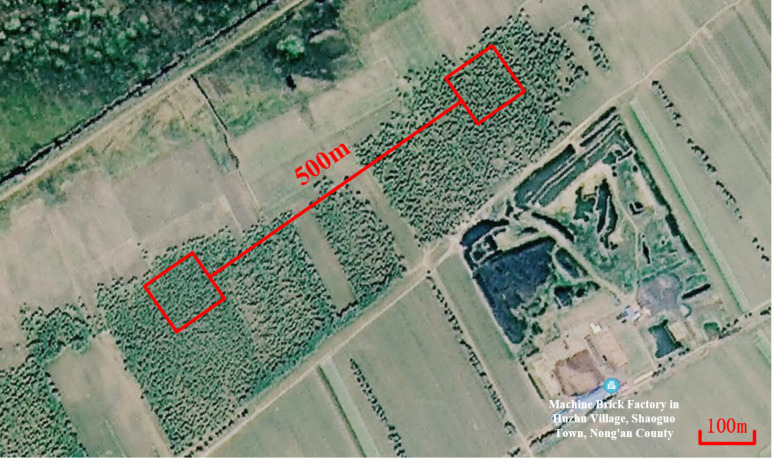
Diagram of MRS dual-system detection experiment.

Generally, dual systems are used to carry out work in conventional field detection projects. Therefore, to verify the effectiveness of the collaborative detection method, two sets of JLMRS-III groundwater detectors are used for field tests near the Taipingchi Reservoir in the suburbs of Changchun. A satellite image of the experimental site is shown in Figure 8. The environmental noise of the site is low, the experimental environment is good, and there are drilling data for comparison and verification. In the experimental process, a single-turn square coil with a side length of 100 m is used, and the transmitting antennas of the two sets of instruments are placed on the ground parallel to one another. The distance between the two nearest parallel sides is 500 m, which ensures that the two sets of instruments do not affect each other when they undergo an alternating operation. The performance parameters of the two systems are shown in Table 1.

**Figure 9:**
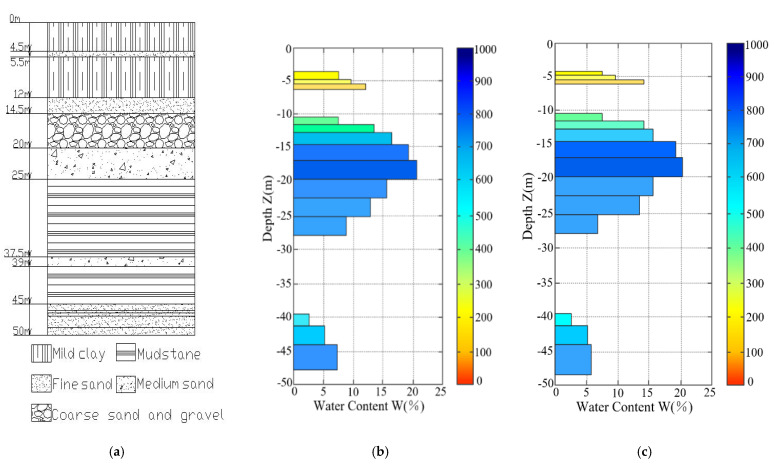
Comparison between the actual measurement and drilling results of the two systems in the survey area. (**a**) Drilling results in the survey area; (**b**) JLMRS_III_1 water content measurement results; (**c**) JLMRS_III_2 water content measurement results.

The test process is as follows: the two instruments begin to undergo an alternating operation. The first MRS instrument transmits the “received” message to the wireless communication system and then to the second MRS instrument’s upper computer control system through the wireless communication system. Then, the host computer control system sends instructions to start operation, and each instrument completes the measurement. By counting the time, we find the individual detection time of each magnetic resonance sounding instrument to be 62 min. It takes 35 min for the two magnetic resonance sounding instruments to work together. The drilling results at the measurement location are shown in Figure 9a. The water content inverted by the detection points of the two sets of instruments is shown in Figure 9b,c. The abscissa represents the water content, and the ordinate represents the depth of underground detection. The color bar on the right side represents the lateral decay time of T_2_*, which reflects the nature of the aquifer medium. Comparing these three pictures together horizontally can intuitively and clearly prove the consistency of the detection results with the real results. 

## 6. Conclusions

We proposed a multisystem joint detection method of magnetic resonance sounding, which effectively improves the detection efficiency of a large-area exploration space through the coordinated operation of two sets of instruments. By studying the electromagnetic interference and coupling characteristics between the two transmitting antennas when the two instruments worked together, we found that when the distance between the transmitting antennas of the two instruments is 30.8 km, the free-detection mode can be implemented at the same time without intersystem communication.

In addition, the proposed method operates on the short-distance scale; that is, when the distance between the transmitting antennas of the two instruments is greater than 300 m, the two instruments can be used in cooperative detection mode to complete the measurement work, and the interference can be reduced through the cooperative work of the instruments. Based on the above detection methods, field test measurements and comparisons were carried out around Changchun. The multisystem joint detection method can decrease the exploration time to half of it.

Through the above two methods, the method and collaborative strategy for the high-efficiency measurement of a magnetic resonance sounding instrument in a fixed detection area are realized. The strategy effectively improves the working efficiency of magnetic resonance sounding technology in a wide range of applications, and it provides strong technical support for improving the applicability and scope of magnetic resonance sounding technology.

## Figures and Tables

**Table 1:** Instrument system performance parameters.

	Name	Parameter
1	System noise floor	1 nV/Hz ^(0.5)^
2	Signal detection sensitivity	0.5 nV
3	Detection system gain range	80–120 dB
4	Filter bandwidth	50 Hz
5	Maximum emission current	200 A
6	Antenna diameter	100 m
